# Identification and Characterization of Two Novel Members of the Family *Eubacteriaceae*, *Anaerofustis butyriciformans* sp. nov. and *Pseudoramibacter faecis* sp. nov., Isolated from Human Feces

**DOI:** 10.3390/microorganisms13040916

**Published:** 2025-04-16

**Authors:** Xiao-Meng Wang, Hao-Jie Huang, Xin-Wei Sun, Rui-Qi Wei, Hao-Yu Chen, Chang Liu, Shuang-Jiang Liu

**Affiliations:** 1 State Key Laboratory of Microbial Technology, Shandong University, Qingdao 266237, China; xxiaomengwang@163.com (X.-M.W.); hhj00123123@163.com (H.-J.H.); xinwei98s@sina.com (X.-W.S.); wrq233627@163.com (R.-Q.W.); chenhaoyu0219@163.com (H.-Y.C.); 2 State Key Laboratory of Microbial Resources, Environmental Microbiology Research Center (EMRC), Institute of Microbiology, Chinese Academy of Sciences, Beijing 100101, China

**Keywords:** *Eubacteriaceae*, *Anaerofustis butyriciformans*, *Pseudoramibacter faecis*, strain HA2171^T^, strain HA2172^T^, polyphasic taxonomy, genomic analyses, metabolic profiles, human gut microbiome

## Abstract

Members of *Eubacteriaceae* are involved in host health and diseases. Two Gram-stain-positive, strictly anaerobic, non-motile, non-spore-forming, and rod-shaped bacterial strains, HA2171^T^ and HA2172^T^, were isolated from the feces of Chinese healthy donors. Based on 16S rRNA gene sequences, HA2171^T^ and HA2172^T^ belonged to the family *Eubacteriaceae*. Physiological and biochemical characterizations indicated that HA2171^T^ and HA2172^T^ were neutrophilic, mesophilic, and tolerant to low-concentration NaCl. The major cellular fatty acids (>10.0%) of HA2171^T^ were C_16:0_, C_14:0_, C_18:1ω7c_, and C_17:0_ 2-OH, and those of HA2172^T^ were C_14:0_ and C_16:0_. MK-6 was the respiratory quinone in both strains. Phylogenetic and phylogenomic analyses showed that HA2171^T^ was closest to *Anaerofustis stercorihominis* ATCC BAA-858^T^ and that HA2172^T^ as closest to *Pseudoramibacter alactolyticus* ATCC 23263^T^. Genome annotation revealed that the HA2171^T^ and HA2172^T^ were able to metabolize carbohydrates and produce acetate and butyrate. HA2172^T^ contains genes associated with hydrogen sulfide production, which is a potential risk for diseases. Based on the phylogenetic, phenotypic, and chemotaxonomic characteristics, we propose that HA2171^T^ and HA2172^T^ represent two novel species, and the names *Anaerofustis butyriciformans* sp. nov. and *Pseudoramibacter faecis* sp. nov. are proposed.

## 1. Introduction

With the advancement of sequencing technology, the relationship between gut microbiota and host health has become increasingly evident. The gut microbiome plays crucial roles in food digestion, training host immunity, regulating gut endocrine function and neurological signaling, modifying drug activity and metabolism, eliminating toxins, and producing numerous compounds that would influence the host [1,2]. The family *Eubacteriaceae*, a group of bacteria that rarely form endospores, is obligatorily anaerobic and Gram-stain-positive, and are frequently small rods (~0.5 μm) [3]. At the time of this article’s writing, the family *Eubacteriaceae* stands for 10 genera and 47 species with validly published names (https://lpsn.dsmz.de/family/eubacteriaceae, accessed on 1 January 2025). The genera *Anaerofustis* and *Pseudoramibacter* of the family *Eubacteriaceae* were isolated from feces samples [4,5]. The genus *Anaerofustis* was first described and validly published by Finegold et al. [4] with a single species (https://lpsn.dsmz.de/genus/anaerofustis, accessed on 1 January 2025), *A*. *stercorihominis* ATCC BAA-858^T^. The genus *Pseudoramibacter* was first described by Willems et al. [6], and two species (*P*. *alactolyticus* ATCC 23263^T^; *Pseudoramibacter porci* DSM 106894^T^) have been validly published [5,6] at the time of the writing of this article (https://lpsn.dsmz.de/genus/pseudoramibacter, accessed on 1 January 2025).

The members of family *Eubacteriaceae* play important and complicated roles in host health and diseases. As a major short-chain fatty acids (SCFAs) producer in the gut, *Eubacteriaceae* species play a key role in maintaining intestinal homeostasis [7,8,9,10]. The relative abundances of *Eubacteriaceae* were reportedly reduced in Crohn’s disease [11], cystic fibrosis [12], and ulcerative colitis [13]. As beneficial to hosts, *Anaerofustis* strains could enhance fiber digestion and the production of SCFAs [14] and potentially enforce intestinal antioxidant properties [15,16]. The genus *Anaerofustis* has also been linked to imbalanced fat metabolism due to its interactions with host genes involved in fat metabolism, ultimately influencing fat deposition in hosts [17]. However, members of the genus *Pseudoramibacter* could be harmful to the host, as they are linked to infections [18] and diseases [19].

Herein, we report the isolation, cultivation, and characterization of strains HA2171^T^ and HA2172^T^. Based on the results of phylogenetic analysis and 16S rRNA gene identity, strains HA2171^T^ and HA2172^T^ were identified as novel species from genus *Anaerofustis* and genus *Pseudoramibacter* in the family *Eubacteriaceae*, for which the names *Anaerofustis butyriciformans* HA2171^T^ and *Pseudoramibacter faecis* HA2172^T^ were proposed, respectively. In in silico analysis, we further investigated the potential associations of these two novel species with a disease. Given the importances of the family *Eubacteriaceae*, the newly described bacterial strains of *Eubacteriaceae* provide resources for understanding their roles in host health and diseases.

## 2. Materials and Methods

### 2.1. Sample Collection and Treatment

Fecal samples were collected from two healthy Chinese volunteers, and their informed consent for fecal sample donation were obtained. The donor’s fresh feces were collected and immediately transferred to an anaerobic workstation (COY-8300600, Coy, Ann Arbor, MI, USA) filled with a gas mix (N_2_: H_2_: CO_2_ = 85:10:5). All experiments were conducted in the anaerobic workstation unless otherwise specified. Fecal samples were first suspended in PBS buffer (P1020, Solarbio, Beijing, China) and then filtered through a 40 μm cell strainer (15-1040, Biologix, Jinan, China) to remove large insoluble particles. The filtrate was serially diluted in PBS in series of 10^−1^ to 10^−7^, and the dilutions of 10^−5^ to 10^−7^ were spread into different agar plates and subsequently incubated at 37 °C anaerobically for 3–15 days.

### 2.2. Culture Media and Preservation

Modified mGAM medium (mmGAM) and modified PYG medium (mPYG) were used for bacterial cultivation and isolation. The mmGAM (pH 7.0) was prepared according to [20], and was sterilized at 115 °C for 25 min. The PYG medium (HB0398, Hopebio, Qingdao, China) was modified by supplementing the following components (per L): 0.5 g of xylose, 0.5 g of L-cysteine hydrochloride, 5 mL of haemin, 1 mL of resazurin, 50 mL of clarified rumen fluid, 50 mL of sheep blood, 5 mL of vitamin K1 solution (HB8462, Hopebio), 1 mL of Wolfe’s vitamin solution (SL0110, Coolaber, Beijing, China), 1 mL of Wolfe’s mineral solution (SL0120, Coolaber), and adjusted to a pH of 7.2, and after sterilization at 115 °C for 25 min, the pH lowered to 6.8. Single colonies were picked, and the pure culture was cultured on mmGAM agar or in liquid medium for enrichment-culturing. Nitrate medium was employed for the nitrate reduction experiment. The nitrate broth was prepared with the following components (per liter of distilled water), 1 g of KNO_3_, 2 g of Na_2_HPO_4_, 1 g of glucose, 20 g of peptone, and 1 g of agar, and adjusted to pH = 7.2. All strains were preserved using three different methods, slant culture, glycerol storage, and freeze-drying, in accordance with the protocol previously described [21]. They were also deposited at the China General Microbiological Culture Collection Centre (CGMCC) and Korean Collection for Type Cultures (KCTC).

### 2.3. Morphological, Physiological and Biochemical Taxonomic Determinations

Colonies of strains HA2171^T^ and HA2172^T^ were grown on mmGAM agar plates for 5 days at 37 °C, and colony morphology was observed with optical microscopy (Y-TV55, Nikon, Tokyo, Japan) and stereoscopic microscopy (DS-F2.5, Nikon). Cellular morphology was observed by transmission electron microscopy (FEI Tecnai G2 F20, Thermo Scientific, Waltham, MA, USA).

Spore formation was examined by optical microscopy (Y-TV55, Nikon) after staining using the Spore Stain Kit (HB8300, Hopebio) and spores were predicted according to methods reported previously [22]. Gram-staining was carried out using a Gram-stain Kit (G1060, Solarbio) and cell motility was examined using optical microscopy (Y-TV55, Nikon). The temperature range for growth was determined at 16, 20, 25, 30, 35, 37, 42, 45, 50, and 65 °C in mmGAM broth for 5 days, and cell growth was estimated by measuring the turbidity at 600 nm (OD_600_) using a UV/visible spectrophotometer (Ultrospec 10, Biochrom, Cambridge, UK). The pH range for growth was tested at pHs of 4.0, 5.0, 5.5, 6.0, 6.5, 7.0, 7.5, 8.0, 9.0, 10.0, and 11.0 in mmGAM broth for 5 days, and a buffer solution for adjusting pH was prepared according to methods reported previously [20]. NaCl tolerance was determined in mmGAM broth supplemented with 0, 0.5, 1.0, 1.5, 2.0, 2.5, 3.0, 3.5, 4.0, 5.0, and 6.0% (*w*/*v*) NaCl. Ox bile powder (8008-63-7, Yuanye, Shanghai, China) tolerance was determined in mmGAM broth supplemented with 0.3% (*w*/*v*). Aerobic growth of the strains was tested in liquid mGAM medium glass tubes and incubated at 37 °C in a shaker at 200 rpm for 5 days. Susceptibility of strains HA2171^T^ and HA2172^T^ to the following antibiotics (Bkmam; μg per disk unless otherwise stated) was examined by the disk diffusion method: chloramphenicol (30), clindamycin (2), erythromycin (15), clarithromycin (15), azithromycin (15), cefixime (5), carbenicillin (100), rifampin (5), ampicillin (100), penicillin (10 IU), ceftriaxone (30), amoxicillin (25), gentamicin (10), streptomycin (10), tetracycline (30), vancomycin (30), ciprofloxacin (5), bacitracin (0.04 IU), cefoperazoneB (300 IU), and kanamycin (30). The diameters of the inhibitory zone were measured after anaerobic cultivation at 37 °C for 48 h. All tests were performed in triplicate.

Other physiological and biochemical analyses were carried out by using the API ZYM identification kit (25200, BioMérieux, Marcy-l′Étoile, France), API 20A identification kit (CN2030025, BioMérieux), Biolog AN identification kit (NO.1007, Biolog, Hayward, CA, USA), nitrate reduction identification kit (HB8282, Hopebio), and Lead acetate test paper (L903523, Macklin, Shanghai, China), according to the manufacturers’ instructions. To determine SCFAs’ production, cells were cultured in mmGAM for 96 h at 37 °C and then were pelleted, and the supernatant was collected. An equal volume of ethyl acetate was added to the supernatant and vortexed to extract the SCFA products. The gas chromatography mass spectrometry (GC-MS) analysis was carried out using an GC-MS-QP2010 Ultra apparatus equipped with an autosampler and a DB-wax capillary column (30 m, 0.25 mm i.d., 0.25 m film thickness, Shimadzu, Santa Clara, CA, USA) [20]. The whole-cell fatty acids, polar lipids, and respiratory quinones were determined according to methods reported previously [20].

### 2.4. Phylogenetic and Genomic Analysis

The 16S rRNA genes of strains were amplified and sequenced with the universal bacterial primers 27F and 1492R [23]. For the identification of bacteria, 16S rRNA gene sequences were aligned on EzBioCloud [24], and the related validly published type strains were downloaded from the GenBank (www.ncbi.nlm.nih.gov/genbank/, accessed on 1 January 2025). Phylogenetic analysis was carried out using MEGA 11 [25] with the neighbor-joining (NJ) [26] maximum-likelihood (ML) [27] and maximum-parsimony (MP) [28]. Phylogenetic trees were generated based on Kimura’s two-parameter method [29]. Branching patterns of the NJ tree were evaluated by bootstrapping with 1,000 replicates. The bootstrap consensus trees resulting from the ML and MP methods supported the NJ tree. The CVTree [30] method was used to construct phylogenomic trees based on whole genomes.

The genomic DNAs of strains HA2171^T^ and HA2172^T^ were extracted with the commercial TIANamp Bacteria DNA Kit (DP302, Tiangen, Beijing, China) following the manufacturer’s instruction. Whole-genome sequencing was performed using the Illumina system at Guangdong Magigene Biotechnology Co., Ltd. (Guangzhou, China) (www.magigen.com, accessed on 1 January 2025). The genomic assembly was performed with the SPAdes software (version 3.9.0) [31]. The values of average nucleotide identity (ANI) [32] and digital DNA-DNA hybridization (dDDH) [33] were calculated by the online tools developed by the ANI Calculator [34] and the Genome-to-Genome Distance Calculator (3.0) [35], respectively. The genomes of *A. stercorihominis* ATCC BAA-858^T^ (GCA_000154825), *P. alactolyticus* ATCC 23263^T^ (GCA_000185505), and *P. porci* RF 744 FAT 4^T^ (GCA_009696145) were downloaded and jointly analyzed using the following software. The genome annotation and pathway analysis of the strain genomes were analyzed using KAAS (http://www.genome.jp/kegg/kaas/, accessed on 1 January 2025) [36]. The carbohydrate-utilizing capacities of the 5 strains were analyzed using their genome data and three tools (HMMER, dbCAN_sub, DIAMOND) in dbCAN3 (https://bcb.unl.edu/dbCAN2/, accessed on 1 January 2025) [37]. All hits were retained for subsequent analysis. If the annotations were identified by one or two tools (considering low confidence) and by all three tools, results are shown in black and colors (Tables S3–S7), respectively. The primary and secondary metabolites of the strains were analyzed using gutSMASH (version 2.0.0) [38] and antiSMASH (version 6.1.1) [39].

## 3. Results

### 3.1. Morphological and Physiological Properties

**Figure 1:**
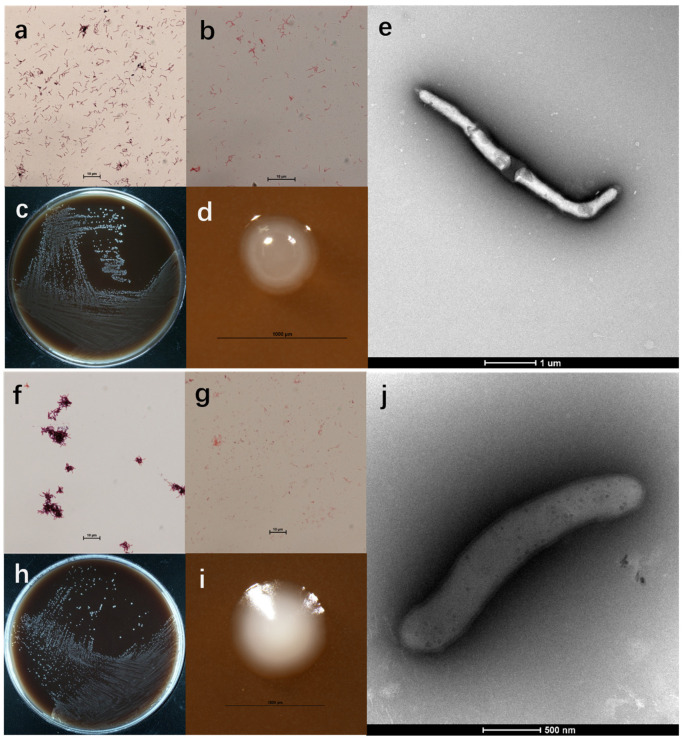
Bacterial cell morphology and colony. Optical graphs of Gram-stained cells (**a**,**b**,**f**,**g**), colonies on mmGAM agar (**c**,**d**,**h**,**i**), and cell morphology under transmission electron microscopy (**e**,**j**). Strain HA2171^T^ (**a**–**e**) and HA2172^T^ (**f**–**j**).

**Figure 2:**
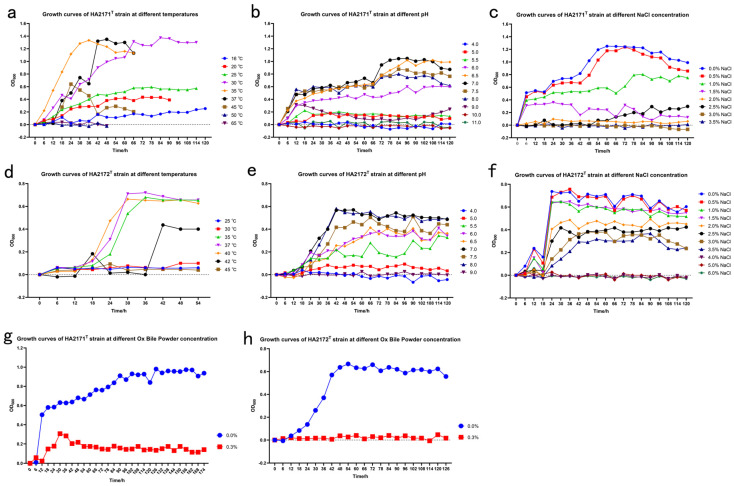
Growth curve of strains HA2171^T^ (**a**–**c**,**g**) and HA2172^T^ (**d**–**f**,**h**) at different temperatures, pHs, and NaCl and Ox bile powder concentrations.

Cells of strain HA2171^T^ were Gram-stain-positive (Figure 1a), non-spore-forming (Table S1 and Figure 1b), non-motile, non-pigmented, and rod-shaped (2.4–4.9 µm long × 0.2–0.3 µm wide) (Figure 1c–e). After anaerobically cultivating on mmGAM agar plates for 5–7 days, colonies on the mmGAM agar plates (0.3–0.5 mm in diameter) appeared as opaque, white in color, and elliptical in shape with a smooth and raised texture, and displayed neat and shiny edges (Figure 1c,d). Growth occurred at temperatures ranging from 16 to 45 °C (optimum temperature 35 °C), the pH range for growth was from 5.5 to 8.0 (optimum pH = 7.0), and the NaCl concentration for growth was from 0.0 to 1.5% NaCl (*w*/*v*) (optimum 0.0% NaCl (*w*/*v*)). Growth was observed for strain HA2171^T^ while incubating at 35 °C for 5 days in the mmGAM liquid medium with 0.3% (*w*/*v*) Ox bile powder (Figure 2a–c,g). This observation suggests that the strain is bile-tolerant, which is likely related to its potential probiotic function. No growth was observed for strain HA2171^T^ while incubating at 35 °C for 5 days in the mmGAM liquid medium with oxygen, indicating that strain HA2171^T^ was strictly anaerobic. The isolation sources and physiological characteristics of strains HA2171^T^ and HA2171^T^ from their phylogenetically close neighbors are summarized in Table 1.

Strains: 1, HA2171^T^ (this study); 2, *Anaerofustis stercorihomini* ATCC BAA-858^T^ [4]; 3, *Alkalibaculum bacchi* CP11^T^ [40]; 4, *Alkalibaculum sporogenes* M08 DMB^T^ [41]; 5, *Alkalibacter mobilis* M17 DMB^T^ [42]; 6, *Irregularibacter muris* 2PG-426-CC-4.2^T^ [43]; 7, HA2172^T^ (this study); 8, *Pseudoramibacter alactolyticus* ATCC 23263^T^ [6]; 9, *Pseudoramibacter porci* DSM 106894^T^, has limited information available [5]; 10, *Eubacterium limosum* ATCC 8486^T^ [3,44,45,46]; 11, *Eubacterium callanderi* FD^T^ [47]; 12, *Eubacterium barkeri* ATCC 25849^T^ [3,48,49]. +, positive; −, negative; W, weakly positive; ND, no data available. Key features that differentiate the studied species in this study from their closely related species are bolded and shown in red.

Cells of strain HA2172^T^ were Gram-stain-positive (Figure 1f), non-spore-forming (Table S1 and Figure 1g), non-motile, non-pigmented, and rod-shaped (2.2–6.7 µm long × 0.3−0.5 µm wide) (Figure 1h–j). After anaerobic cultivation on mmGAM agar plates for 5–7 days, colonies (0.5–1.0 mm in diameter) appeared as opaque, white in color, elliptical in shape with a smooth and raised texture, and displayed neat and shiny edges (Figure 1h,i). Growth occurred at temperatures ranging from 20 to 45 °C (optimum temperature 37–40 °C), the pH range for growth was from 5.5 to 8.0 (optimum pH 7.0), the and the NaCl concentration for growth was from 0.0 to 3.5% NaCl (*w*/*v*) (optimum 0.0% NaCl (*w*/*v*)). No growth was observed for strain HA2172^T^ while incubating at 37 °C for 5 days in the mmGAM liquid medium with 0.3% (*w*/*v*) Ox bile powder (Figure 2d–f,h). No growth was observed for strain HA2172^T^ with oxygen. The isolation sources and physiological characteristics of strains HA2171^T^ and HA2171^T^ from their phylogenetically close neighbors are summarized in Table 1.

### 3.2. Chemotaxonomic Characteristics

**Figure 3:**
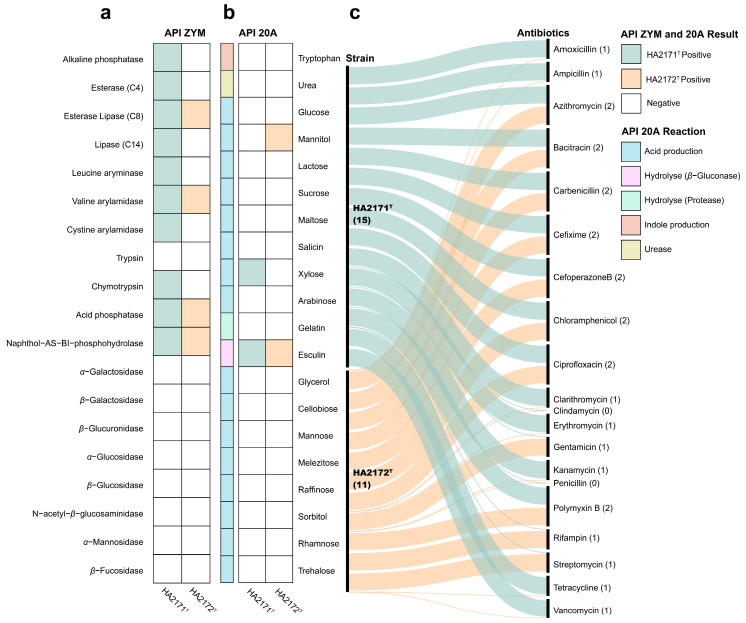
API ZYM test results for enzyme activities (**a**), API 20A test results for biochemical characteristics of anaerobic bacteria (**b**), and the evaluation of antibiotic resistance (**c**) of strains HA2171^T^ and HA2172^T^. For (**a**), with the color green and orange representing the ability of enzymes for substrates conversion activity, while white represents the inability to convert them. For (**b**), the color green and orange represent the ability to utilize substrates for acid production, indole production, hydrolysis reaction, and urease activity, while white represents the inability to utilize them. For panel (**c**), this section shows the resistance of the strains to 20 antibiotics, with the number of strains in parentheses.

The API ZYM test (Figure 3a) revealed that strain HA2171^T^ was different from *A. stercorihominis* ATCC BAA-858^T^ in the enzymatic reaction of the substrate from esterase, esterase lipase, naphthol-AS-Bi-phosphohydrolase, alkaline phosphatase, valine arylamidase, and cystine arylamidase, but there were similarities between those of trypsin, catalase, *β*-glucosidase, or *N*-acetyl-*β*-glucosaminidase. Strain HA2172^T^ promotes the enzymatic reactions of substrates such as esterase lipase and naphthol-AS-Bi-phosphohydrolase, while no data were available for *P. alactolyticus* ATCC 23263^T^. The API 20A test (Figure 3b) revealed that strain HA2171^T^ was different from *A. stercorihominis* ATCC BAA-858^T^ in the fermentation production of acids from glucose and xylose but there were similarities in maltose, lactose, mannitol, mannose, or sucrose. Strain HA2172^T^ was different from *P. alactolyticus* ATCC 23263^T^ in the fermentative production of acids from glucose but there were similarities in those from mannitol, xylose, glycerol, cellobiose, maltose, mannose, sucrose, or lactose. The resistance of strains HA2171^T^ and HA2172^T^ to 20 antibiotics was tested and they both are resistant to chloramphenicol, cefoperazoneB, ciprofloxacin, azithromycin, bacitracin, cefixime, carbenicillin, and polymyxin B, but susceptible to penicillin and clindamycin (Figure 3c). The Biolog AN test (Table S2) revealed that strain HA2171^T^ is positive for adonitol, D-arabitol, D-cellobiose, dextrin, dulcitol, i-erythritol, D-fructose, D-galactose, D-galacturonic acid, *α*-D-glucose, glucose-6-phosphate, glycerol, lactulose, maltotriose, D-mannose, D-melibiose, 3-melthyl-D-glucose, D-raffinose, D-sorbitol, stachyose, sucrose, turanose, glyoxylic acid, L-phenylalanine, L-valine, L-valine plus L-aspartic acid, 2′-deoxy adenosine, thymidine, and uridine. Strain HA2172^T^ is positive for D-galactose, D-galacturonic acid, gentiobiose, glucose-6-phosphate, D-mannose, 3-melthyl-D-glucose, palatinose, and glyoxylic acid. The distinctive biochemical characteristics of strains HA2171^T^ and HA2171^T^ from their phylogenetically close neighbors are summarized in Table 1. All biochemical tests were performed in triplicate.

**Figure 4:**
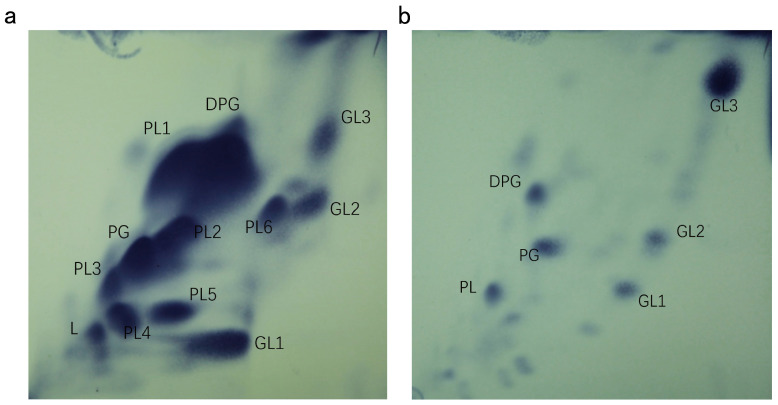
Polar lipids profile of strains HA2171^T^ (**a**) and HA2172^T^ (**b**) following two-dimensional TLC and stained with molybdatophosphoric acid. DPG, diphosphatidylglycerol; PG, phosphatidylglycerol; GL1-3, unidentified glycolipids; PL1-6, unidentified phospholipid.

The cellular fatty acids of *A. stercorihominis* ATCC BAA-858^T^ were determined after cultivation in BKV medium [4], but strain HA2171^T^ did not grow in BKV broth. Strain HA3432, isolated from our laboratory collection, exhibited a 16S rRNA gene sequence similarity of 99.5% to *A*. *stercorihominis* ATCC BAA-858^T^. We cultivated strain HA2171^T^ and strain HA3432 in mmGAM broth. Cells were harvested and subjected to cellular fatty acid profiling. The predominant cellular fatty acids (>10%) of strain HA2171^T^ were C_16:0_ (19.9%), C_14:0_ (15.2%), C_18:1*ω*7c_ (12.5%), and C_17:0_ 2-OH (12.1%), while those of strain HA3432 were C_16:0_ (28.8%), C_18:0_ (16.7%), and C_18:1*ω*9c_ (12.6%). The polar lipid profile of strain HA2171^T^ was determined to contain diphosphatidylglycerol, phosphatidylglycerol, three unidentified glycolipids, and six unidentified phospholipids (Figure 4a). The respiratory quinone of strain HA2171^T^ was menaquinone MK-6.

The predominant cellular fatty acids (>10%) of strain HA2172^T^ were C_14:0_ (49.0%) and C_16:0_ (22.0%). There is currently no data available of fatty acid information for *P. alactolyticus* ATCC 23263^T^. Another member of the *Pseudoramibacter* genus, *P*. *porci* DSM 106894^T^, had C_14:0_ (32.0%), C_16:0_ (14.0%), and iso/anteiso-C_17:1_ (29.0%) as its main cellular fatty acid in GAM medium [5]. Long-chain fatty acids (C14–C16) are an important component of cell membrane lipids and their anabolism is involved in maintaining the structure and function of cell membranes [50,51]; the high proportion of C_14:0_ suggests a preference for lipid metabolism and is preferentially accumulated. The polar lipid profile of strain HA2172^T^ was determined to contain diphosphatidylglycerol, phosphatidylglycerol, three unidentified glycolipids and one unidentified phospholipid (Figure 4b). The respiratory quinone of strain HA2172^T^ was menaquinone MK-6.

### 3.3. Phylogeny and Phylogenomic Features

**Figure 5:**
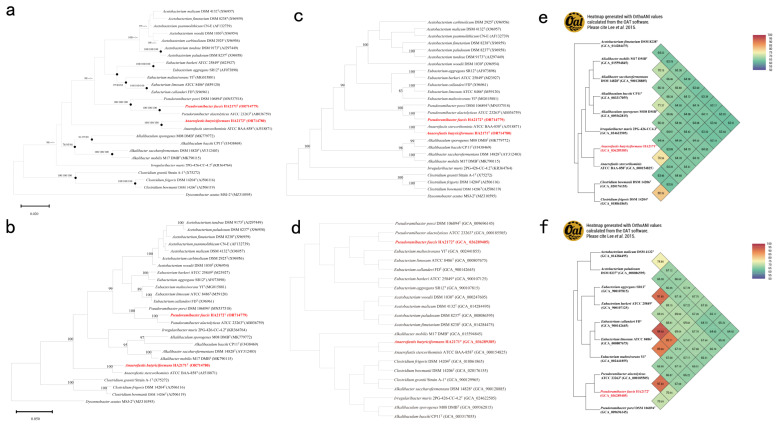
Phylogenetic analysis of strains HA2171^T^, HA2172^T^, and their closely related type strains within the family *Eubacteriaceae* [32]. For (**a**), a phylogenetic tree constructed with the NJ method based on the 16S rRNA sequences of strains HA2171^T^, HA2172^T^, and the closely related type strains within the family *Eubacteriaceae*. *Dysosmobacter acutus* MSJ-2^T^ was used as an outgroup. Sequences were downloaded from GenBank. Filled circles indicate branches that were also found in trees generated with the ML and MP algorithms. Numbers at branch nodes represent confidence levels (values > 70% are shown) from 1000 replicate bootstrap samplings, followed by the number of NJ/ML/MP. For (**b**,**c**), phylogenetic trees of HA2171^T^ and HA2172^T^ based on the 16S rRNA gene constructed with maximum-likelihood (**b**) and maximum parsimony (**c**) methods, *Dysosmobacter acutus* MSJ-2^T^ was set as the outgroup. Numbers at branch nodes represent confidence levels (only values ≥ 70% are shown) from 100 replicate bootstrap samplings. Displayed in d is a phylogenomic tree of HA2171^T^ and HA2172^T^ (**d**), and a phylogenomic tree of strains HA2171^T^ and HA2172^T^ displaying the positions of HA2171^T^, HA2172^T^, and their phylogenetically closely related neighbors (top 10 to HA2171^T^, the range of the 16S rRNA gene identity was 87.6–97.1%; and top 10 to HA2172^T^, the range of the 16S rRNA gene identity was 90.6–97.7%). e and f display ANI heat maps based on whole genomes of HA2171^T^ (**e**) and HA2172^T^ (**f**). GenBank accession numbers of the genomes are shown in parentheses. The red portion of the figure highlights the two novel species identified in this study.

Strain HA2171^T^ was phylogenetically closest to *A. stercorihominis* ATCC BAA-858^T^ (Figure 5a), their 16S rRNA gene similarity was 97.1% and their average nucleotide identity (ANI) [34] was 76.6% (Figure 5e). The ML and MP tree viewing (Figure 5b,c), as well as phylogenomic analysis (Figure 5d) revealed that strain HA2171^T^ was a member of the genus *Anaerofustis*. The digital DNA-DNA hybridization (dDDH) value between the genome of strain HA2171^T^ and *A. stercorihominis* ATCC BAA-858^T^ was 21.2%. The Percentage of Conserved Proteins (POCP) value between strain HA2171^T^ and *A. stercorihominis* ATCC BAA-858^T^ was 68.0%. Considering that the ANI and dDDH values of strain HA2171^T^ were lower than 95.0% and 70.0%, respectively, the threshold values for species delineation [52], and the POCP values were higher than the thresholds of 50.0% for bacterial genus classification [43,53], we proposed that strain HA2171^T^ represented a novel species within the genus *Anaerofustis*.

The phylogenetic analysis based on 16S rRNA gene sequences revealed that strain HA2172^T^ was closest to *P. alactolyticus* ATCC 23263^T^ (Figure 5a–c), with the similarity of 97.7% and their ANI was 91.8% (Figure 5f). Phylogenomic analysis (Figure 5d) suggested that strain HA2172^T^ was a member of the genus *Pseudoramibacter*. The dDDH value between the genome of strain HA2172^T^ and *P. alactolyticus* ATCC 23263^T^ was 46.2%. The POCP value between strain HA2172^T^ and *P. alactolyticus* ATCC 23263^T^ was 79.6%. Due to this, the ANI and dDDH values of strain HA2171^T^ and strain HA2172^T^ were lower than 95.0% and 70.0%, the threshold values for species delineation [52]. The POCP value were higher than the thresholds of 50.0% for bacterial genus classification [43,53]; thus, we proposed that strain HA2172^T^ represented a novel species within the genus *Pseudoramibacter*.

### 3.4. In Silico Analysis of Biological Characterization and Gut-Associated Functional Potential

#### 3.4.1. Taxonomic Differentiation

The genome size of strain HA2171^T^ was 2,187,946 bp, which harbored 2205 coding sequences, including 46 tRNA genes, 5 rRNA genes (containing 1 16S rRNA gene), and 2 sRNA. The total length of these coding regions accounted for 88.6% of the genome. The size of the strain HA2172^T^ genome was 2,337,197 bp, which harbored 2529 coding sequences, including 49 tRNA, genes and 7 rRNA genes (which contain 1 16S rRNA gene). The total length of these coding regions accounted for 87.6% of the genome. The genome G + C contents of strains HA2171^T^ and HA2172^T^ were 52.0% and 29.8%, respectively, which fell within the G + C content range of 27.1–70.0 mol% for the family *Eubacteriaceae* [4,54].

#### 3.4.2. Metabolic Potential

**Figure 6:**
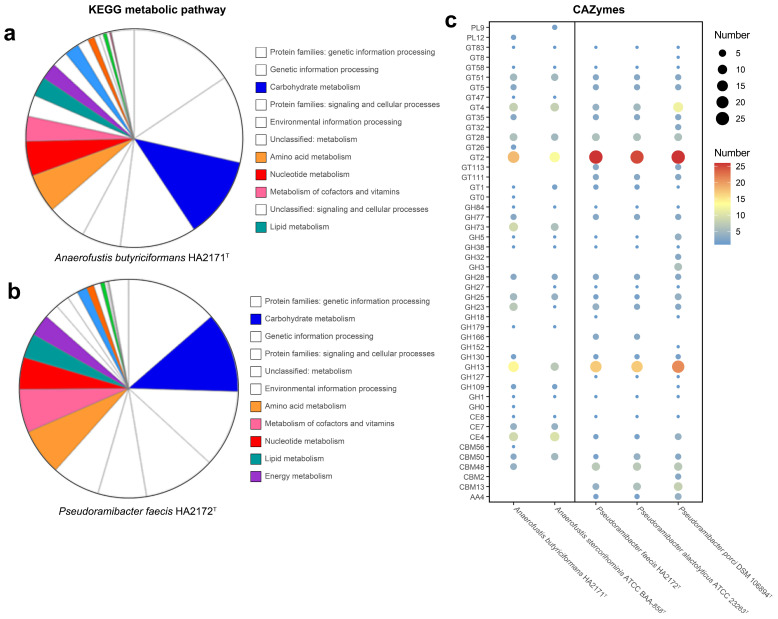
KEGG metabolic pathway of strains HA2171^T^ (**a**) and HA2172^T^ (**b**). Distribution of carbohydrate utilization-related genes in the genomes of strains HA2171^T^, HA2172^T^, and closely related species (**c**). For (**c**), the circle size corresponds to the number of CAZymes, with larger circles indicating a greater number.

Gene functions analyzed with KEGG indicated that the majority of the genes of strain HA2171^T^ were related to carbohydrate metabolism (134), amino acid metabolism (64), nucleotide metabolism (57), metabolism of cofactors, and vitamins (41) and lipid metabolism (32). Compared with *A. stercorihominis* ATCC BAA-858^T^, strain HA2171^T^ had fewer genes related to carbohydrate metabolism (134 vs. 171). According to KEGG annotation results (Figure 6a), HA2171^T^ could metabolize a carbohydrate into pyruvate and further into acetic acid. Gene functions analyzed with KEGG indicated that the majority of the genes of strain HA2172^T^ were related to carbohydrate metabolism (150), amino acid metabolism (87), metabolism of cofactors, and vitamins (81), nucleotide metabolism (60) and lipid metabolism (46). Strains HA2172^T^ and *P. alactolyticus* ATCC 23263^T^ had similar functional gene compositions, such as carbohydrate metabolism (150 vs. 146), amino acid metabolism (87 vs. 88), metabolism of cofactors, vitamins (81 vs. 84), nucleotide metabolism (60 vs. 57), and lipid metabolism (46 vs. 49). According to KEGG annotation results (Figure 6b), HA2172^T^ could metabolize a carbohydrate into pyruvate and further into acetic acid.

The gut microbiota plays a critical role in providing energy to the host by facilitating the breakdown and metabolism of dietary fibers, thereby influencing overall health. We investigated the differences in carbohydrate metabolism capabilities between the strains HA2171^T^ and HA2172^T^, as well as their closely related species. Our results demonstrate that HA2171^T^ harbors a significantly greater number of carbohydrate-active enzymes (CAZymes) compared to *A. stercorihominis* ATCC BAA-858^T^ (125 vs. 87) (Figure 6c). This disparity is largely attributed to the presence of additional GH77, GH130, and PL12 families, as well as an increased number of GH13 and GH23 enzymes, which are predominantly involved in the degradation of starch [55], mannans [56], heparin [57], and peptidoglycans [56]. While, HA2172^T^ exhibits a CAZyme profile more closely resembling that of *P. alactolyticus* ATCC 23263^T^, with a notably lower CAZyme count compared to *P. porci* DSM 106894^T^ (114, 113 vs. 153) (Figure 6c). This difference is mainly due to the absence of GH3 and a reduced representation of GH5, with related cellulases [58,59]. Notably, HA2172^T^ retains GH152 laminarinase activity [60]. The observed differences in CAZymes’ composition between HA2172^T^ and *P. porci* DSM 106894^T^ are likely influenced by host-specific factors. Notably, we retained all annotations by three dbCAN3 tools (Tables S3–S7), and those annotations that were identified by one tool might be less accurate. Thus, a Supplementary Table is provided, showing those annotations by both the NCBI non-redundant protein database (nr) and the CAZy database (Table S8).

The primary and secondary metabolites produced by microorganisms are key factors influencing the gut microbiota. Analysis using gutSMASH version 2.0.0 revealed that HA2171^T^ and *A. stercorihominis* ATCC BAA-858^T^ share similar primary metabolites, with both capable of converting pyruvate into formate, acetate, and butyrate (Table S9). HA2172^T^ produced a broader range of primary metabolites, and its metabolic profile was very similar to that of *P. alactolyticus* ATCC 23263^T^, which also demonstrated the ability to convert pyruvate to formate, acetate, and butyrate. Additionally, HA2172^T^ is involved in branched-chain amino acid debranching, producing branched-chain fatty acids (Table S9). Notably, HA2171^T^ produced H_2_S based on our test results but was not predicted from genome annotation (Table S9), while HA2172^T^ did not produce H_2_S. The productions of H_2_S and branched-chain fatty acids have been reported as potential risk factors for intestinal immune disorders, such as Crohn’s disease [61,62]. Furthermore, secondary metabolites predicted by antiSMASH version 6.1.1 revealed that strain HA2171^T^ contains genes involved in ribosomal modification, including ranthipeptides and non-ribosomal peptide synthetases (NRPS) (Table S10).

#### 3.4.3. Implications for Human Health

SCFAs are primarily generated through the fermentation of dietary fiber by gut microbes [63], playing a crucial role in mediating direct and indirect interactions within the microbiota [64]. The major fermentative products of strain HA2171^T^ were acetic acid (1.8 μg mL^−1^) and butyric acid (20.9 μg mL^−1^). The major products of strain HA2172^T^ fermentation were acetic acid (1.6 μg mL^−1^), butyric acid (8.5 μg mL^−1^), and valeric acid (2.8 μg mL^−1^). Notably, the butyrogenic capacity of HA2171T was moderate compared to established butyrate producers such as *Faecalibacterium prausnitzii* [65] and *Eubacterium rectale* [66]. These metabolites exhibit clinically relevant physiological effects: butyrate serves as the primary energy substrate for colonic epithelial cells while exerting anti-inflammatory [67], anticancer [68], and gut barrier-enhancing activities [69]. It is of particular interest that HA2172^T^-produced valerate has been implicated in protection against nonalcoholic fatty liver disease (NAFLD)-associated hepatocellular carcinoma [70] and demonstrates antimicrobial and anti-inflammatory properties that contribute to metabolic homeostasis [71]. Similarly, acetate participates in energy homeostasis [72] and cholesterol metabolism [73], while also displaying antimicrobial activity [74]. Collectively, these findings underscore the functional divergence between these strains, suggesting their potential niche-specific roles in shaping gut microbiota and host physiology.

## 4. Discussion

The family *Eubacteriaceae* is a member of the *Bacillota* phylum, mainly consisting of Gram-positive, anaerobic, or facultative anaerobic bacteria [3]. This family is found in various environments, especially in the human gut, which plays an important role in maintaining intestinal health and metabolic processes. In this study, two novel members in the genera *Anaerofustis* and *Pseudoramibacter* of *Eubacteriaceae* have been discovered and described. Based on 16S rRNA gene sequence results and phylogenetic analysis, strains HA2171^T^ and HA2172^T^ belonged to the genera *Anaerofustis* and *Pseudoramibacter*, respectively. According to previous reports, the abundance of *Anaerofustis* is positively correlated with mortality in antibiotic-associated diarrhea [75], and *Anaerofustis* has been observed to be significantly enriched in the gut microbiota of patients with inflammatory bowel disease (IBD) [76]. *Pseudoramibacter* has been frequently detected in root canal samples and is associated with oral infections such as pulpitis and periapical lesions [18,77]. Additionally, based on a population cohort analysis, it is suggested that the decreased abundance of *Pseudoramibacter* is associated with depression in elderly Chinese patients with functional constipation [78]. These findings suggest that members of these two genera may pose potential risks to human health.

To reveal the evolutionary and functional differences in novel species, comparison with their closest species is crucial. In physiological and biochemical experiments, strains HA2171^T^ and *A. stercorihominis* ATCC BAA-858^T^ show differences in the enzymatic activities of alkaline phosphatase, naphthol-AS-Bi-phosphohydrolase, valine arylamidase, cystine arylamidase, esculin hydrolysis, glucose, and xylose utilization. Similarly, HA2172^T^ and *P. alactolyticus* ATCC 23263^T^ show differences in their esculin hydrolysis and glucose utilization, the utilization of other substrates or the same enzyme activity (Table 1). Microbial metabolites play a crucial role in shaping the composition and function of the gut microbiota, thereby influencing host health and disease. These metabolites, including SCFAs [64], bile acids [79], and various other organic compounds, exert their effects through multiple mechanisms that modulate microbial interactions and host responses. GC-MS analysis indicated that the predominant fermentation products of strain HA2171^T^ were acetic acid and butyric acid. In contrast, strain HA2172^T^ exhibited a broader profile of fermentation products, including acetic acid, butyric acid, and valeric acid. Genomic analysis revealed results that are consistent with experimental observations, demonstrating that both strains harbor metabolic pathways for the conversion of pyruvate into acetate and butyrate. This metabolic capability underscores their potential to produce key SCFAs.

A striking discordance was observed between genome annotation and phenotypic assays regarding hydrogen sulfide (H_2_S) production. Notably, strain HA2171^T^ demonstrated H_2_S production capability despite the absence of detectable H_2_S synthesis-related genes in its genome. Conversely, strain HA2172^T^ lacked measurable H_2_S production activity but harbored a genome annotation for H_2_S synthesis pathways, mirroring its closest relative *P*. *alactolyticus* ATCC 23263^T^ [6]. This genotype–phenotype discrepancy underscores the limitations of solely relying on genomic predictions. H_2_S production has been implicated as a potential risk factor for intestinal immune diseases such as IBD [80], irritable bowel syndrome (IBS) [81], and colorectal cancer (CRC) [82]. As a gaseous signaling molecule, H_2_S regulates the onset and progression of IBD in the host by modulating the host inflammatory response [83,84], oxidative stress [84,85], intestinal barrier function [86,87], and the balance of the gut microbes [86]. At low concentrations, H_2_S regulates the release of inflammatory mediators by inhibiting inflammatory signaling pathways such as nuclear factor *κ*B (NF-*κ*B), regulates redox balance by scavenging reactive oxygen species (ROS) and enhancing antioxidant enzyme activities [84], maintains the intestinal barrier integrity by upregulating the expression of tight junction proteins such as Zonula Occludens-1 (ZO-1) and occludin [87], and regulates microbiota homeostasis by influencing the composition of the gut microbes and the metabolic activities to regulate microbiota homeostasis. However, in the patient with IBD and IBS, the increased abundance of opportunistic pathogens, such as sulfate-reducing bacteria (SRB), may lead to higher levels of biogenic H_2_S production, which can damage colonocytes, compromise the intestinal barrier, and result in increased gut microbes translocation, thereby promoting sustained inflammatory responses [80]. In addition, H_2_S effects symptoms such as abdominal pain, diarrhea, or constipation in patients with IBS by modulating intestinal motility, visceral sensitization [88], and gut microbial composition [81]. Meanwhile, H_2_S also interferes with host regulatory mechanisms by modulating the tumor microenvironment [89] and other key biological processes to influence CRC occurrence, progression, and metastasis. The results outlined above highlight the need for further validation of genome analysis findings through the isolation and cultivation of strains.

The difference in carbohydrate metabolism ability has an important influence on the ecological adaptability [90], physiological function [91], and interaction with metabolites [92]. The comparative analysis of the CAZyme profiles of strains HA2171^T^ and HA2172^T^ provides valuable insights into their distinct CAZyme capabilities and potential ecological roles. Strain HA2171^T^ exhibits a unique CAZyme profile compared to *A. stercorihominis* ATCC BAA-858^T^, primarily due to the presence of additional GH77, GH130, and PL12 families, as well as an increase in GH13 and GH23 enzymes. These enzymes are crucial for the degradation of complex carbohydrates such as starch [55], mannans [56], heparin [57], and peptidoglycans [56], which indicates that HA2171^T^ has colonization potential in populations characterized by gut microbiota dysbiosis, a high-fiber dietary intake, and metabolic syndrome disease. The presence of these specific CAZymes suggests that HA2171^T^ has evolved to efficiently utilize a diverse range of carbohydrate substrates, which may provide it with a competitive advantage in its ecological niche. In contrast, strain HA2172^T^ displays a CAZyme profile that is more similar to *P. alactolyticus* ATCC 23263^T^, with significantly lower CAZyme counts compared to *P. porci* DSM 106894^T^. This difference is primarily attributed to the absence of GH3 and a reduced representation of GH5 cellulases. These findings highlight the importance of CAZyme diversity in microbial adaptation and function. The distinct CAZyme profiles of HA2171^T^ and HA2172^T^ reflect their unique metabolic capabilities and potential roles in carbohydrate metabolism within their respective environments. Future research should focus on elucidating the specific mechanisms by which these CAZymes contribute to microbial fitness and host interactions, as well as exploring the ecological and evolutionary factors driving these differences. Understanding these mechanisms will provide deeper insights into the functional diversity of microbial communities and their impact on host health and environmental processes.

## 5. Conclusions

Based on the phylogenetic and phenotypic characteristics, we concluded that strains HA2171^T^ and HA2172^T^ represented novel species of the genera *Anaerofustis* and *Pseudoramibacter*, for which the names *Anaerofustis butyriciformans* sp. nov. and *Pseudoramibacter faecis* sp. nov. are proposed. Genomic analyses have revealed distinct differences in their metabolic profiles and ecological niche adaptations, providing a theoretical foundation for discussing the potential impact of these members on human diseases.

## 6. Descriptions

### 6.1. Description of Anaerofustis butyriciformans sp. nov.

*Anaerofustis butyriciformans* (bu.ty.ri.ci.for’mans. N.L. neut. n. *acidum butyricum*, butyric acid; L. pres. part. *formans*, forming; N.L. part. adj. *butyriciformans*, producing butyric acid).

Cells are Gram-stain-negative, anaerobic, non-motile, non-spore-forming, non-pigmented, and rod-shaped (2.4–4.9 µm long × 0.2–0.3 µm wide). Colonies on mmGAM agar are opaque, white, elliptical, smooth, and raised, with neat and shiny edges, and about 0.3–0.5 mm in diameter. Growth is observed within a temperature range of 16–45 °C (optimum at 37 °C), a pH range of 5.5–8.0 (optimum at 6.5–7.0), and a NaCl concentration range of 0.0–1.5% NaCl (*w*/*v*) (optimum at 0.0% NaCl (*w*/*v*)). It produces acetic acid and butyric acid. Indole is not produced, with no urease activity. H_2_S can be produced. Nitrate reduction is absent, and the catalase is negative. It hydrolyzed esculin and gelatin. Acids are produced by fermenting glucose and xylose, but not tryptophan, urea, mannitol, lactose, saccharose, maltose, salicin, arabinose, glycerol, cellobiose, mannose, melezitose, raffinose, sorbitol, rhamnose, or trehalose. Strain HA2171^T^ is positive for alkaline phosphatase, esterase, esterase lipase, leucine aryminase, acid phosphatase, and naphthol-AS-BI-phosphohydrolase, while it is negative for trypsin, *α*-galactosidase, *β*-galactosidase, *β*-glucuronidase, *α*-glucosidase, *β*-glucosidase, N-acetyl-*β*-glucosaminidase, *α*-mannosidase, and *β*-fucosidase. It assimilates adonitol, D-arabitol, D-cellobiose, dextrin, dulcitol, i-erythritol, D-fructose, D-galactose, D-galacturonic acid, *α*-D-glucose, glucose-6-phosphate, glycerol, lactulose, maltotriose, D-mannose, D-melibiose, 3-melthyl-D-glucose, D-raffinose, D-sorbitol, stachyose, sucrose, turanose, glyoxylic acid, L-phenylalanine, L-valine, L-aspartic acid, 2′-deoxy adenosine, thymidine, and uridine. The predominant cellular fatty acids of strain HA2171^T^ are C_16:0_, C_14:0_, C_18:1*ω*7c_, and C_17:0_ 2-OH. The polar lipids include diphosphatidylglycerol, phosphatidylglycerol, three unidentified glycolipids and six unidentified phospholipids. The respiratory quinone is menaquinone MK-6.

The G + C content is 52.0 mol%. The type strain HA2171^T^ (=CGMCC 1.18050^T^ = KCTC 25721^T^) was isolated from the feces of a healthy adult.

### 6.2. Description of Pseudoramibacter faecis sp. nov.

*Pseudoramibacter faecis* (fae’cis. L. gen. n. *faecis*, of feces, after the inoculum source of the strain).

Cells are Gram-stain-positive, anaerobic, non-motile, non-spore forming, non-pigmented, and rod-shaped (2.2–6.7 µm long × 0.3–0.5 µm wide). Colonies on mmGAM agar are opaque, white, oval shaped, smooth and raised, with neat edges and luster, and are about 0.5–1.0 mm in diameter. Growth is observed within a temperature range of 20–45 °C (optimum at 42 °C), a pH range of 5.5–8.0 (optimum at 7.0) and a NaCl concentration range of 0.0–3.5% NaCl (*w*/*v*) (optimum at 0.0% NaCl (*w*/*v*)). It produces acetic acid, butyric acid, and valeric acid. Indole is not produced, with no urease activity. It does not produce H_2_S. Nitrate reduction is absent, and the catalase test result is negative. It hydrolyzed esculin and gelatin. Acids are produced by fermenting mannitol, but not tryptophan, urea, glucose, xylose, lactose, saccharose, maltose, salicin, arabinose, glycerol, cellobiose, mannose, melezitose, raffinose, sorbitol, rhamnose, or trehalose. Strain HA2172^T^ is positive for acid phosphatase and naphthol-AS-BI-phosphohydrolase, but negative for alkaline phosphatase, esterase, lipase, leucine aryminase, cystine arylamidase, trypsin, chymotrypsin, *α*-galactosidase, *β*-galactosidase, *β*-glucuronidase, alpha-glucosidase, *β*-glucosidase, N-acetyl-*β*-glucosaminidase, *α*-mannosidase, and *β*-fucosidase. It assimilates D-galactose, D-galacturonic acid, gentiobiose, glucose-6-phosphate, D-mannose, 3-melthyl-D-glucose, palatinose, and glyoxylic acid. The predominant cellular fatty acids were C_14:0_ and C_16:0_. The polar lipids include diphosphatidylglycerol, phosphatidylglycerol, three unidentified glycolipids, and one unidentified phospholipid. The respiratory quinone is menaquinone MK-6.

The G + C content is 29.8 mol%. The type strain HA2172^T^ (=CGMCC 1.18049^T^ = KCTC 25722^T^) was isolated from the feces of a healthy adult.

## Figures and Tables

**Table 1:** Differential characteristics of strains HA2171^T^ and HA2172^T^ compared to their close relatives of the family *Eubacteriaceae*.

	1	2	3	4	5	6	7	8	9	10	11	12
**Isolation sources**	Human feces	Human feces	Livestock-impacted soil	Terrestrial mud volcano	Coastal lake	Muris of a mouse	Human feces	Dental calculus	Aachen minipig	Human feces	Anaerobic digestor	Human feces
**Motility**	−	ND	+	+	+	ND	−	−	ND	−	−	−
**Aesculin/Esculin hydrolysis**	**+**	**−**	−	ND	ND	+	**+**	**−**	ND	+	−	+
**Acid production from:**												
Glucose	**+**	**W**	+	+	+	−	**−**	**+**	ND	+	+	+
Xylose	**+**	**W**	−	+	−	−	−	−	ND	W	−	−
Glycerol	−	ND	−	−	−	−	−	−	ND	−	+	+
Cellobiose	−	ND	−	−	−	−	−	−	ND	−	−	−
Maltose	−	−	−	−	−	−	−	−	ND	−	−	−
Mannitol	−	−	−	ND	ND	−	+	+	ND	+	ND	+
Mannose	−	−	+	+	+	−	−	−	ND	−	−	−
Sucrose	−	−	−	−	+	−	−	−	ND	−	ND	−
Lactose	−	−	−	−	−	−	−	−	ND	−	−	−
**Enzyme activities:**												
Esterase C4	**+**	**W**	W	ND	ND	ND	−	ND	ND	ND	ND	ND
Esterase lipase C8	**+**	**W**	−	ND	ND	ND	+	ND	ND	ND	ND	ND
Trypsin	−	−	−	ND	ND	ND	−	ND	ND	ND	ND	ND
Catalase	−	−	ND	−	ND	ND	−	ND	ND	−	ND	ND
*β*-Glucosidase	−	−	−	ND	ND	ND	−	ND	ND	ND	ND	ND
Alkaline phosphatase	**+**	**−**	−	ND	ND	ND	−	ND	ND	ND	ND	ND
Naphthol-AS-Bi-phosphohydrolase	**+**	**W**	W	ND	ND	ND	+	ND	ND	ND	ND	ND
*N*-Acetyl-*β*-glucosaminidase	−	−	W	ND	ND	ND	−	ND	ND	ND	ND	ND
Valine arylamidase	**W**	**−**	−	ND	ND	ND	−	ND	ND	ND	ND	ND
Cystine arylamidase	**W**	**−**	−	ND	ND	ND	−	ND	ND	ND	ND	ND
**DNA G + C content (mol%)**	52.0 mol%	70.0 mol%	34.0 mol%	32.3 mol%	39.1 mol%	35.8 mol%	29.8 mol%	61.0 mol%	49.3 mol%	47.2 mol%	47.0 mol%	45.0 mol%

## Data Availability

All data generated or analyzed from this study are included in this published article. The GenBank/EMBL/DDBJ accession number for the 16S rRNA gene sequence of strain HA2171^T^ is OR714780 and that of strain HA2172^T^ is OR714779. This whole genome shotgun project of strains HA2171^T^ and HA2172^T^ has been deposited at DDBJ/ENA/GenBank under the accession JAYFFH000000000 and JAYFFI000000000, respectively. The version described in this paper is version JAYFFH010000000 and JAYFFI010000000.

## Supplementary Materials

The following supporting information can be downloaded at: https://www.mdpi.com/article/10.3390/microorganisms13040916/s1, Table S1: Summary of 66 conserved genes linked to the sporulation capabilities in strains HA2171^T^ and HA2172^T^; Table S2: The Biolog AN test results for carbon sources’ assimilation in strains HA2171^T^ and HA2172^T^; Tables S3–S7: The distribution of carbohydrate utilization-related genes in the genomes of strains HA2171^T^, HA2172^T^, and closely related species were analyzed using three dbCAN3 tools; Table S8: Genome annotations of HA2171^T^, HA2172^T^, and related species were validated using NCBI nr and CAZy databases; Table S9: Summary of primary metabolite gene clusters in HA2171^T^, HA2172^T^ and closely related species; Table S10: Summary of secondary metabolite gene clusters in HA2171^T^, HA2172^T^, and closely related species.
